# A Method of Detecting Lacerations of the Cervix Uteri Post Partum

**Published:** 1885-09

**Authors:** John Bartlett

**Affiliations:** North Clark Street


THE CHICAGO

MEDICAL JOURNAL AND EXAMINER.

VOL. LI. SEPTEMBER, 1885. No. 3.

### ORIGINAL COMMUNICATIONS.

### A METHOD OF DETECTING LACERATIONS OF THE CERVIX UTERI POST PARTUM. *By* JOHN BARTLETT, M. D.

Read before the Chicago Medical Society, Aug. 3rd, 1885.)

My object in addressing the society this evening is to suggest a way and a time in which laceration of the cervix uteri may be easily and certainly detected soon after its occurence. Directly after delivery, if the fingers be introduced deeply into the vagina up to the contracted os uteri internum, and then carried in any direction a little outwardly, the flabby and floating ring formed by the non-contractile cervix may be felt, as Guillemeau described it, three hundred years ago, "like a section of large intestine." By very carefully following the entire circumference of this ring, an existing rent may be discovered. But this examination is attended with some difficulties. The patient is exhausted with her labor, and fatigued with our attentions, and just now, since "it is all over," longing for rest. She is impatient of, and perturbed by, this post-factum inquiry. Her state of mind, and possible expression of complaint, are apt to render an examination, which the physician cannot regard as absolutely necessary, less exact and thorough than it would be otherwise. And then, the soft and floating margins of the cervix uteri have often somewhat of an intangible feel, if I may so express myself, gliding past the fingers like a detached clot of blood, and occasionally, in some portion of their circumference, passing out of satisfactory reach.

On these accounts it is not surprising to hear an obstetrician say that he cannot tell whether the post partum cervix is lacerated or not. Now, I desire to teach those who may not be familiar with the short lesson that I propose to impart this evening, how to discover a cervixal laceration after labor. The error of accoucheurs who fail to recognize such a condition is that they do not make their observation of the suspected cervix at the proper time. They examine the neck actually as we have just done mentally—after the clearance of the uterus. The favorable moment for the examination—and this is the gist of my remarks—is just as the placenta is beginning to occupy and distend the cervix. The collar of flesh is then not floating and uncertain, but on the stretch, expanded, forming a distinct ring easily followed in its entire circumference. At this moment, then, just as the cervical tube is being rendered tense by the placental mass, any laceration in it may be detected with ease and certainty.*

North Clark Street.

---

*Dr. Bartlett illustrated his remarks with earthenware models, turned by a potter under his immediate direction.

